# Development of SSR Markers Based on Transcriptome Sequencing and Association Analysis with Drought Tolerance in Perennial Grass *Miscanthus* from China

**DOI:** 10.3389/fpls.2017.00801

**Published:** 2017-05-16

**Authors:** Gang Nie, Lu Tang, Yajie Zhang, Linkai Huang, Xiao Ma, Xin Cao, Ling Pan, Xu Zhang, Xinquan Zhang

**Affiliations:** Department of Grassland Science, Animal Science and Technology College, Sichuan Agricultural UniversitySichuan, China

**Keywords:** bio-fuel, RNA sequencing, markers development, association analysis, *Miscanthus sinensis*

## Abstract

Drought has become a critical environmental stress affecting on plant in temperate area. As one of the promising bio-energy crops to sustainable biomass production, the genus *Miscanthus* has been widely studied around the world. However, the most widely used hybrid cultivar among this genus, *Miscanthus × giganteus* is proved poor drought tolerance compared to some parental species. Here we mainly focused on *Miscanthus sinensis*, which is one of the progenitors of *M. × giganteus* providing a comparable yield and well abiotic stress tolerance in some places. The main objectives were to characterize the physiological and photosynthetic respond to drought stress and to develop simple sequence repeats (SSRs) markers associated with drought tolerance by transcriptome sequencing within an originally collection of 44 *Miscanthus* genotypes from southwest China. Significant phenotypic differences were observed among genotypes, and the average of leaf relative water content (RWC) were severely affected by drought stress decreasing from 88.27 to 43.21%, which could well contribute to separating the drought resistant and drought sensitive genotype of *Miscanthus*. Furthermore, a total of 16,566 gene-associated SSRs markers were identified based on Illumina RNA sequencing under drought conditions, and 93 of them were randomly selected to validate. In total, 70 (75.3%) SSRs were successfully amplified and the generated loci from 30 polymorphic SSRs were used to estimate the genetic differentiation and population structure. Finally, two optimum subgroups of the population were determined by structure analysis and based on association analysis, seven significant associations were identified including two markers with leaf RWC and five markers with photosynthetic traits. With the rich sequencing resources annotation, such associations would serve an efficient tool for *Miscanthus* drought response mechanism study and facilitate genetic improvement of drought resistant for this species.

## Introduction

As a result of global climate change, drought has become a critical environmental factor negatively affecting on plant growth, development, survival as well as crop yield (Boyer, 1982; Seki et al., 2007; Shinozaki and Yamaguchi-Shinozaki, 2007; Maughan et al., 2012; Malinowska et al., 2017). The genus *Miscanthus* is a perennial rhizomatous C4 grass which widely recognized as a promising bio-energy crops to sustainable biomass production (Hodkinson et al., 2002; Somerville et al., 2010). Despite the water use efficiency of C4 crops is superior to C3 crops (Gowik and Westhoff, 2011; Ings et al., 2013), water availability still limits the maximum yields achievable. *Miscanthus × giganteus*, crossing between *Miscanthus sacchariflorus* and *Miscanthus sinensis*, is the most widely used hybrid cultivar among this genus in Europe and North America as a biomass feedstock (Greef et al., 1997; Hodkinson et al., 2002; Heaton et al., 2008, 2009; Hastings et al., 2009). However, owing to its natural sterility and a narrow genetic base, it is difficult to improve *M. × giganteus* with desirable trait through breeding and just one genotype is available for agricultural production in most areas (Nishiwaki et al., 2011; Dwiyanti et al., 2014) Furthermore, poor WUE of *M. × giganteus* had been reported compared to some parental species (Clifton-Brown and Lewandowski, 2000). As a progenitor of *M. × giganteus*, new varieties *M. sinensis* widely adapted to a wide range of geographical conditions (Clifton-Brown and Lewandowski, 2002; Nie et al., 2016) and showed extensive genetic diversity, which is desirable to provide improved drought tolerance and comparable biomass yield. Compared with other C4 grasses like maize (*Zea mays* L.) and Saccharum (*Saccharum officinarum* L.), *M. sinensis* has a relatively high efficient photosynthesis and water use efficiency (Greef et al., 1997; Long and Spence, 2013) owing to its perennial growth habit and extensive root system (Byrt et al., 2011; van der Weijde et al., 2013).

In order to maximize the avoidance competition of arable land with major grain crops (Quinn et al., 2015; van der Weijde et al., 2016), most non-food grasses like *Miscanthus* were commonly planted in vast marginal land, which always limited water supply, although drought or water deficit always affects crop yield seriously worldwide (Cattivelli et al., 2008). Generally, wild plants showed high tolerant performance against abiotic stresses (Ellis et al., 2000; Bartels, 2001; Mittova et al., 2004), but it may not necessarily favor growth or biomass accumulation under stress rather survival. For *M. sinensis*, the attractive characteristics is efficient biomass production rather than just survival and yield is always strongly linked to water availability. Therefore, stabilizing crop performance under drought stress, increasing crop potencial production of per unit of water consumption, will become a critical goal for *M. sinensis* breeding (Ings et al., 2013).

Previous study had been reported that *M. × giganteus* showed poorly responds to low water availability (Ings et al., 2013). van der Weijde et al. (2016) evaluated the growth and quality of 50 diverse *Miscanthus* genotypes under drought stress and proved that genotypic variation for drought stress exist in this genus. Malinowska et al. (2017) used a high-throughput phenomics facility to screen 47 *Miscanthus* genotypes about biomass accumulation and water use under drought stress. All the related researches had offered valuable insights into the varied side of drought-induced responses and the growth as well as yield characteristics of *Miscanthus* under drought stress. However, despite an increasing concern about the drought resistant of *Miscanthus*, little is known about the physiological and photosynthetic mechanisms response to drought stress.

In addition, the type and timing of physiological or photosynthetic changes induced upon drought stress always vary in different species and genotypes (Centritto et al., 2009; Costa et al., 2012). The conventional method for screening genotypes with desirable traits is time-consuming and labor intensive (Furbank and Tester, 2011). Thereby, it is necessary to develop efficient methods for screening the favorable traits associated with drought resistance around the wide range *Miscanthus* population. Molecular markers are essential tools for estimating genetic variation, selecting parental or breeding line and marker-assisted breeding for improvement of crop desirable traits (Hung et al., 2009; Clark et al., 2014; Nie et al., 2014, 2016; Liu et al., 2015). The number of available efficient molecular markers for *Miscanthus* is still not enough, especially the function markers associated with drought tolerance. SSRs are valuable molecular markers for most plants that genome sequence is not available and have been widely used for molecular MAS of major crop plants. Compared with traditional marker development methods depending on the publicly available cDNA and genomic libraries, recent advances high-throughput next-generation RNA sequencing (NGS) platform provide a high-efficiency tool for developing large amounts of gene-based SSRs markers in most crop species (Hu et al., 2004; Gupta and Prasad, 2009; Varshney, 2009) and proved powerful associated with putative functions involving varies abiotic stress tolerance and important agronomic traits (Kane and Rieseberg, 2007; Yu et al., 2011; Nie et al., 2016).

In this study, the main objective was to characterize the physiological and photosynthetic respond to drought stress with a population of 44 wild collections of *Miscanthus*. Mapping the drought resistant mechanism of *Miscanthus* could help us to well predict and assess desirable traits of the crops in extreme environments. Furthermore, the development of SSRs markers associated with drought tolerance by transcriptome sequencing of *Miscanthus* will facilitate the speed of the screening across the genotypes, which aimed to provide a platform for *M. sinensis* genetic improvement and molecular marker-assisted breeding.

## Materials and Methods

### Plant Material and Drought Treatment

A total of 44 diverse *Miscanthus* genotypes comprising 41 *M. sinensis* (NO. 1 to NO. 41), 2 *Miscanthus floridulus* (NO. 48 to NO. 49) and 1 *M. × giganteus* (NO. 50) species used in this study were mainly collected from the Southwest of China (Supplementary Table S1). Each genotype was propagated to six individuals (three for control and three for treatment) by dividing rhizome and grown in plastic pots (20 cm × 25 cm) with mixture soil (50% loam with 50% fine sandy). All materials were grown in greenhouse with a completely randomized design from April to July in 2014. Before subjected to water stress, all the plants grew with sufficient conditions. The experiment was designed to evaluate the genotypic difference in physiological and photosynthetic aspect responding to water stress. Prior to treat, all the replicates were well watered with the average of 90% SWC measured by Soil Moisture Equipment TDR300 (Santa Barbara, CA, USA). Naturally water stress was applied by stopping water for 30 days. The photosynthetic traits were measured at the end of the drought stress in control and treatment first, and then leaf samples were collected for determination physiological indexes from three replications.

### Physiological and Photosynthetic Measurements

The Pn, Gs, Ci, and Tn were determined by photosynthetic system (LI-6400, Li-Cor, Lincoln, NE, USA) with the CO_2_ concentration was 400 μmol mol^-1^ and photosynthetic active radiation (PAR) was 1,200 μmol⋅m^-2^s^-1^. Gs and Ci were measured under the condition that with a saturated light intensity of 1,000 μmol m^-2^s^-1^. Ten randomly selected leaves were measured to determine related traits for each replicate (Zhao et al., 2014).

The RWC of leaves was measured by drying method (Barrs and Weatherley, 1962). Approximate 0.2 g (FW) fresh leaves randomly selected from each pot were wrapped in absorbent paper and put into the 50mL tube with filled water. Tubes were placed in the dark place for 24 h, and then take out the leaves, dry the surface moisture, and weight SW. Followed the leaf samples were placed in the oven with 75°C for 3 days to constant weight, weight DW. The RWC was calculated as follows: RWC (%) = (FW - DW/SW - DW) × 100. For the extraction of MDA, a total of 0.1 g leaf samples were quickly frozen in liquid nitrogen (2 min), sufficiently grind in 2 volume solutions containing 50 mM phosphate buffer (pH = 7.8) and 1% (w/v) polyvinyl polypyrrolidone. The supernatant was then collected after the homogenate centrifuged at 12,000 rpm for 30 min and the content of MDA was examined (Dhindsa and Matowe, 1981).

Analysis of variance of the physiological and photosynthetic index was conducted by using SPSS 17.0 software (IBM, Armonk, NY, USA) and the Pearson correlation analysis of all traits was performed. Effects of both treatment and genotypes were determined by using the model of Least Significant Difference test (LSD).

### Identification and Validation of SSRs

A total of six RNA samples from *M. sinensis* under drought stress were extracted based on the manufacturer’s instructions of the RNeasy Plant Mini Kit (Qiagen, Valencia, CA, USA). RNA purity, concentration, and integrity were checked by using the RNA Nano 6000 Kit for the Agilent 2100 Bioanalyzer 2100 System (Agilent Technologies, USA). The qualified samples were primary stored at –80°C after isolation and quality assessment. Each cDNA library was constructed using 5 μg of total RNA and six cDNA sequencing libraries of *M. sinensis* were constructed using the NEB Next^®^ Ultra TM RNA Library Prep Kit for Illumina (New England Bio-labs, USA). Initially, the total RNA was treated with RNase-free DNase I (NEB) for 30 min at 37°C and Poly (A) mRNA was isolated using poly-T oligo-linked magnetic beads. After purification, the poly (A)-containing mRNA was fragmented into 200–250 bp pieces using fragment buffer (Ambion), and the first-strand cDNA was synthesized using random hexamer primers and the short fragments as templates. Then the RNase H and DNA polymerase I were applied to the products for second-strand cDNA synthesizing (16°C for 2 h). Finally, before hybridization, NEB Next Adaptor with hairpin loop structure was ligated to the cDNA and the 3′ ends of the DNA fragments were adenylated. Subsequently, the purity and quality of cDNA fragments library were evaluated using the Agilent Bioanalyzer 2100 system. After generating the clusters on a cBot System using the TruSeq PE Cluster kit v3-cBot-HS (Illumina), Illumina sequencing (paired-end technology in Illumina HiSeq 2000 platform) of the six libraries was performed for RNA-seq analysis. The *de novo* assembly of RNA-seq was conducted using Trinity^1^ with employing the sequence splicing and redundancy removing. In total, 114,747 assembled unigenes were directly used to identify SSRs based on the software MISA^2^ (MIcroSAtellite). SSR search criteria were conducted based on perfect mono-, di-, tri-, tetra-, penta-, and hexa-nucleotide motifs minimum number of six, five, five, four, and four repeats, respectively. Primer3 v2.23 was used to design primers in the flanking regions of the SSRs.

A total of 93 SSR markers (**Table 1**) were randomly selected to validation in 54 plant materials including 44 *M. sinensis*, 5 *M. floridulus*, 1 *M. sacchariflorus*, 1 *M. × giganteus*, and 3 *Hemarthria* cultivars (Supplementary Table S1). The genomic DNA was extracted from the young leaves using plant DNA Genomic Extraction Kit (Tiangen Biotech, China). PCR amplifications were performed in 20 μl total volume reactions, including 3 μl genomic DNA (15 ng/μl), 0.4 μl Golden DNA Polymerase, 10 μl 2X Reaction Mix, 0.5 μl of each primer, and 5.6 μl ddH_2_O. The PCR program was as follows: an initial denaturation at 94°C for 1 min, followed by 5 cycles of 1 min annealing at 60°C, and 1 min extension at 72°C, then followed by 35 cycles of 1 min denaturation at 94°C, 1 min annealing at 55–58°C, and 1 min extension at 72°C. A final 10 min extension at 72°C was used before the PCR reactions were completed. The obtained PCR products were separated on 6% PAGE under 350 volts for 2 h and gel was stained by AgNO_3_ solutions.

**Table 1:** The SSRs primer information identified for validation in this study.

Primer	ID and SSR nr.	SSR	Forward primer	Reverse primer
SAUM-2	CL10031.Contig3_All_5291_1	CAG(3^∗^6)	AGTCTACGGCTACTCCTCCGAT	TGACAAGCTGGGTAGGATATACG
SAUM-3	CL10059.Contig1_All_5294_1	ACC(3^∗^5)	ACAGGGTCTCCCTTGAGTCAG	AACTGGGGAAGAAGACAAGAGAG
SAUM-10	CL10112.Contig3_All_5301_4	AG(2^∗^7)	GTACTCTCTTGCCTTTCCCCTAA	ATTTTGTAACAACACTGCCTGGT
SAUM-17	CL10164.Contig1_All_5323_2	AGGGG(5^∗^4)	ACACCAGCCACATAAGACATTTT	TACCTGAAGCTCTTGATTGCTTT
SAUM-24	CL10242.Contig1_All_5337_1	TCC(3^∗^6)	AGGCTCCTACTTCGCGGTAT	TACAGATCTTGGGGGTTATAGCA
SAUM-28	CL10270.Contig2_All_5347_1	CTT(3^∗^7)	CTGCAGATTCGTCCGAAGG	CTAAGGAGCTGTTCAAGACGAAA
SAUM-41	CL10454.Contig2_All_5376_1	GCT(3^∗^5)	GCCATGTGCAAGAAGGCG	ACCAGGACCTGCACTGGA
SAUM-53	CL10592.Contig1_All_5404_1	TC(2^∗^6)	TTAAACAAGGAACGTCGTCTAGC	TGAAATGTGCCAATAACAATACG
SAUM-54	CL10594.Contig1_All_5405_1	CGCTTC(6^∗^4)	AAAGTTAATGTCGTTGTCGCTGT	ATTTCAGGATCCAAGAGTAAGGC
SAUM-58	CL10635.Contig3_All_5411_1	GT(2^∗^6)	GATCTGGTTTCTGACACTTGCAC	CCGGATTCCAGAGCTTATTCTTA
SAUM-73	CL10784.Contig3_All_5453_1	TA(2^∗^6)	AGGGAGGTATGGATTAACTTGGA	CATTACACAAAATTCTGCACCAA
SAUM-82	CL10854.Contig1_All_5470_1	TA(2^∗^6)	GCGCAAAAAGATTTTCAGTAAAG	TACATAGTACTCCTGCTCGCTCC
SAUM-85	CL10926.Contig2_All_5485_1	TGT(3^∗^6)	AACTTGCTTGGGACTGATAGGAT	ATACTTTCCATGGTGCACAAGAT
SAUM-88	CL1095.Contig2_All_1480_1	CGAGC(5^∗^4)	TAGCTTACGAGCTAAACGAGTCC	GGTTTACAAGCCAACCATGAGTA
SAUM-92	CL1101.Contig3_All_1496_1	AAG(3^∗^5)	CCAATCTACTCCTGTGATTCCAC	AGCTGTTGCCTTCTGTAGCTTTT
SAUM-99	CL11150.Contig1_All_5515_1	AT(2^∗^7)	CTCACCTTCGACCTGAGAAAGTA	TAGTTAAAACGTCCAAGGGTTCA
SAUM-108	CL11268.Contig1_All_5542_3	TTC(3^∗^5)	TCCACAGTACCAACAATTTCAGA	GTTGAGATCACCAGGACAAAGTC
SAUM-111	CL11292.Contig2_All_5550_1	ACG(3^∗^5)	CACCACCGCATATATTATCATCA	CTGTTCTCGCAAAGATCCAAC
SAUM-121	CL11502.Contig2_All_5573_1	TGA(3^∗^6)	AATGATGACGTGGCAGATAAACT	CATCACCATCCACTTCAACACTA
SAUM-128	CL1155.Contig9_All_1528_4	TCG(3^∗^5)	GTAATGGTACAGTACAGGCGGG	ATGAACTACCTCCGCCCAG
SAUM-134	CL11630.Contig7_All_5586_1	GATGG(5^∗^5)	CTCAAAGGTGCAACATTCCTTT	ACCTCCCTCCCTTCCTCC
SAUM-157	CL11875.Contig1_All_5629_1	CCA(3^∗^6)	TTAAATTGATAGAAAGGCCCCAT	CTGACCTAGCATTTCTACCATGC
SAUM-158	CL11881.Contig1_All_5631_1	GCC(3^∗^5)	TAACCCATGGTGTACATGCACTT	AACTTGCCAAAATGACCTAAATG
SAUM-170	CL12059.Contig2_All_5665_1	ATT(3^∗^7)	GCTTAGCTATGATGGTCTCAGGA	TCATCAATTAGCAGCACCTAACA
SAUM-173	CL12095.Contig1_All_5672_1	CTC(3^∗^5)	GATGATTATGATCTTGCTCTCCC	CTGCAATATTCGTGATTGACCTC
SAUM-175	CL12117.Contig2_All_5676_1	GTC(3^∗^5)	GGCTCTTCGTCTCTGTGTACAAT	TCATCCTAACCCTCCAATCTAGC
SAUM-176	CL12143.Contig3_All_5679_1	AT(2^∗^6)	GCTGCAATGTTCACACAAGATAC	GCAAAGGGAACAAGTACAACAAC
SAUM-181	CL1219.Contig3_All_1601_2	AC(2^∗^7)	AGATCGCCAATTCGATTCATAG	TTTCGACTTGGAGAGAAATGTGT
SAUM-198	CL12505.Contig1_All_5730_1	GA(2^∗^10)	GCTCAGATTAGAGAGGGAGAAGG	TCGAAGTGAGAAAATAACACCGT
SAUM-200	CL12623.Contig1_All_5750_1	AGCACC(6^∗^4)	CATCTAATTACACGCCTCCTCAG	GAGACGGTTTCTCTAGGTCGATT

### Population Structure, Clustering, and Association Analysis

Population structure analysis was performed using STRUCTRE v2.3.4 software (Pritchard et al., 2000) with the “admixture model”. Briefly, the software default model parameters were set with 10,000 iterations and 10,000 replications of MCMC. 20 independent runs were applied in this study with varying the assumed number of genetic groups (*K*) from one to six for each run. Maximum likelihood and delta *K* (Δ*K*) values were used to determine the optimum number of subgroups (Pritchard et al., 2000; Evanno et al., 2005). In clustering analysis, the UPGMA dendogram were construct by using similarity coefficients based on SAHN module in the NTSYS-pc version 2.10 software.

The association analysis was conducted by using TASSEL 2.1 software to reveal the association between drought related traits and marker alleles (Bradbury et al., 2007). Four models (simple linear, Q, K, and Q+K) were tested by using Quantile–quantile (QQ) plots to identify the best model fitting drought tolerance related traits for association mapping in *M. sinensis* populations. The significant threshold for selecting the associations between alleles and traits was set at *P* < 0.01. Phenotypic variation explained (*R*^2^) indicated the fixed marker effects.

## Results and Discussion

### Physiological and Photosynthetic Respond

Relative water content determines plant water status when exposed to drought stress. As a physiological indicators that directly response to the extent of plant water deficiency in drought environments, all plants demonstrated high level of leaf RWC in control conditions with an average RWC of 88.27% while evident effect in drought stress with a decrease to 43.21% (**Table 2**). The leaf RWC significantly difference among genotypes based on ANOVA analysis under drought stress which ranged from 12.44 to 66.11%, indicating that leaf RWC could well separate the drought resistant and drought sensitive genotype of *Miscanthus*. The MDA content directly reflected the stability of cellular membrane in the leaves. After a period of drought stress treatment, the MDA content largely increased than control from 11.71 to 54.63 nmol/g FW and showed an extensive variation under drought conditions (19.62–143.78 nmol/g). For the photosynthetic productivity, Pn, Gs, Ci, and Tn were all decreased under drought stress, and significant differences among genotypes were observed under control and drought stress (**Table 2**).

**Table 2:** The minimum, maximum, mean, standard deviation (SD), and *F*-value of RWC, MDA, Pn, Gs, Ci, and Tn under control (*C*) and drought stress (*D*) of *Miscanthus* population.

	RWC (%)		MDA		Pn		Gs		Ci		Tn	
	C	D	C	D	C	D	C	D	C	D	C	D
Min	72.70	12.44	2.83	19.62	1.20	0.06	0.01	0.01	66.13	31.30	0.55	0.28
Max	95.87	66.11	28.14	143.78	26.17	11.36	0.18	0.15	373.44	269.00	11.97	1.95
Mean	88.27	43.21	11.71	54.63	7.72	3.46	0.05	0.03	174.66	123.75	2.06	0.95
SD^a^	4.70	11.28	4.94	24.25	6.16	2.87	0.03	0.02	62.15	45.53	1.58	0.372
*F*	10.15ˆ**	49.41ˆ**	24.01ˆ**	39.54ˆ**	192.68ˆ**	112.40ˆ**	140.86ˆ**	153.97ˆ**	47.25ˆ**	25.38ˆ**	94.74ˆ**	46.28ˆ**

*^∗∗^Significant difference at the 0.01 level.*

As an undomesticated new bioenergy crop, several characteristics related to drought tolerance successfully distinguish *Miscanthus* from many other crops. Since high biomass yield is the primary goal by meeting *Miscanthus* when all above ground biomass is harvested annually, the drought tolerance assessment criterion seems clearly establishment. However, for optimum and stable productivity, at least a 3 years establishment is required after transplanting of *Miscanthus*, which is a typical perennial grass species (Clifton-Brown and Lewandowski, 2000; Anzoua et al., 2015; Nie et al., 2016). Directly evaluating the biomass productive of *Miscanthus* in the field is time and labor consuming under drought conditions. Greenhouse experiment is favorable for detecting the mechanism responding to drought stress which can be identified some reliably measured trait to predict future performance for *M. sinensis* resistant breeding programs.

Leaf RWC reflecting the plant water status, MDA content representing the extent of cellular membrane peroxidation and Pn, Gs, Ci, and Tn comprehensively showing the plant biosynthesis and catabolism process, which provide rapid and easy measurements for screening drought-tolerant plant materials and characterize drought tolerance response mechanism (O’Neill et al., 2006; Jansen et al., 2009; Jiang et al., 2009). Significant correlations were observed between Pn, Gs, Ci, and Tn under well-watered and drought conditions, respectively (**Table 3**). As expect, Pn significant positively correlated with Gs and Tn, and negatively correlated with Ci under drought stress. Leaf RWC significantly correlated with Pn, Gs, Ci, and Tn under well-watered conditions, but such correlations were not observed under drought stress. MDA had no significant correlation with any other measured traits under control, but significantly correlated with RWC under drought stress. The correlation analysis results showed that there is no significant correlation between the physiological and photosynthetic responds to drought stress, indicating that varied and different regulating mechanism was existed in the panel of *Miscanthus* accessions.

**Table 3:** Correlation coefficients among RWC, MDA, Pn, Gs, Ci, and Tn under control and drought stress in *Miscanthus* population^‡^.

	RWC-C	RWC-D	MDA-C	MDA-D	Pn-C	Pn-D	Gs-C	Gs-D	Ci-C	Ci-D	Tn-C
RWC-D	0.071	1									
MDA-C	0.229	-0.050	1								
MDA-D	0.189	-0.500ˆ**	0.239	1							
Pn-C	-0.368ˆ*	0.126	-0.087	0.101	1						
Pn-D	-0.324ˆ*	0.157	0.024	-0.066	0.731ˆ**	1					
Gs-C	-0.523ˆ**	0.117	-0.171	-0.091	0.318ˆ*	0.148	1				
Gs-D	-0.275	0.137	-0.160	0.029	0.761ˆ**	0.663ˆ**	0.041	1			
Ci-C	-0.094	-0.060	-0.252	-0.017	-0.255	-0.464ˆ**	0.381ˆ*	-0.217	1		
Ci-D	0.018	0.181	-0.053	-0.199	-0.377ˆ*	-0.450ˆ**	0.077	-0.335ˆ*	0.576ˆ**	1	
Tn-C	-0.561ˆ**	0.173	-0.249	-0.087	0.543ˆ**	0.310ˆ*	0.941ˆ**	0.300ˆ*	0.268	-0.049	1
Tn-D	-0.158	0.141	-0.033	-0.331ˆ*	0.213	0.533ˆ**	-0.012	0.377ˆ*	-0.396ˆ**	-0.383ˆ*	0.109

*^∗^Correlation is significant at the 0.05 level.*

*^∗∗^Correlation is significant at the 0.01 level.*

*^‡^Correlation calculated using mean of three replicates.*

In order to avoid damage caused by drought stress, one hand the plant reduced water transpiration and improved water use efficiency to protect the photosynthetic mechanism through stomatal closure, on the other hand, adjusted osmotic pressure to increase water uptake and maintained the integrity of the cell structure and function (Yu et al., 2013). In this study, a significant correlation between RWC and MDA content were detected under drought condition and the decreasing of RWC and increasing of MDA content significantly different from that of the control, potentially indicating the occurrence of drought stress, suggesting that they are sufficient parameters of drought tolerance in greenhouse experiment. Generally, drought induced ABA synthesis, which causing stomatal closure to prevents water loss through transpiration and play an important role in maintaining leaf water status (Yu et al., 2013). Previously studies had been showed that *M. × giganteus* exhibited little stomatal regulation ability under mild drought stress compared to *M. sinensis* (Clifton-Brown and Lewandowski, 2000). However, the significant correlation between RWC and Pn, Gs, Ci, or Tn were just detected in well watered condition in this study, indicating that *Miscanthus* holding moisture could not through the stomatal closure pathway but antioxidant pathways to maintain the cell structure and function under drought stress.

In addition, different phenotypic responses of *Miscanthus* to drought stress are important for marker-trait association. The extensive variation observed among the evaluated genotypes regarding physiological and photosynthetic respond indicating that the large range of comprehensive drought tolerance among the evaluated genotypes. The genotypes tested may become important candidates for investigating of mechanisms of plant respond to drought stress and could possibly be used in breeding programs. However, the potential variability in short-term versus long-term drought response should be taken into account in order to further develop new varieties of drought tolerant.

### Identification and Validation of SSRs

Simple sequence repeats are considered as one of the most useful molecular markers due to their broad range of applications in genotype identification, genetic map construction, and marker-trait association. Using the Illumina HiSeq 2000 platform, a total of 349,393,396 raw reads were generated (accession numbers: SRP095822 in the NCBI SRA database) and a total of 316,200,846 high-quality clean reads were assembled into 114,747 unigenes after filtering and trimming the raw reads. Using MISA script, the microsatellites were searched across all unigenes and a total of 16,566 SSRs were identified with motif lengths ranging from one to six bp (**Table 4**). The tri-nucleotide repeats, di-nucleotide repeats and mono-nucleotide repeats were the three most abundant SSRs types in this study and were responsible for 54.63%, 23.84% and 12.81% of the total amount of SSRs, respectively (**Table 4**). However, the mono-nucleotides were excluded in the subsequent analysis owing to the presence of homopolymer affecting the sequencing quality (Gilles et al., 2011). The number of the given repeat unit of SSRs ranged from 4 to >10 and with the number of repeat units increased, the frequency of the given SSR structure progressively decreased (**Table 5**). As for the two most abundant repeat motif types (di- and tri-nucleotides), the frequency of AG/TC motif type account for 52.00% in di-nucleotide repeat motifs, and that the frequency of CCG/GGC was the most abundant motif type in the tri-nucleotide, accounting for 41.80% (**Table 6**).

**Table 4:** Summary of SSRs identified from the combined *M. sinensis*.

SSR information	Number
Total number of sequences examined	114,747
Total number of identified SSRs	16,566
Number of SSR containing sequences	14,272
Number of sequences containing more than 1 SSR	1,959
Number of SSRs present in compound formation	699
Mono-nucleotide repeats	2,122
Di-nucleotide repeats	3,948
Tri-nucleotide repeats	9,051
Quad-nucleotide repeat	288
Penta-nucleotide repeats	510
Hexa-nucleotide repeats	647

**Table 5:** Summary information on frequencies of different SSR repeat motif types related to variation of repeat unit numbers in *M. sinensis*.

Motif length	Repeat unit numberM								
	4	5	6	7	8	9	10	>10	Total
Di	–	–	1,735	869	488	236	265	355	3,948
Tri	–	6,066	2,180	710	91	3	1	–	9,051
Tetra	–	194	93	1	–	–	–	–	288
Penta	457	52	1	–	–	–	–	–	510
Hexa	645	2		–	–	–	–	–	647
Total	1,102	6,314	4,009	1,580	579	239	266	355	14,444
%	7.77	43.92	27.89	10.94	4.03	1.66	1.85	2.50	–

**Table 6:** Statistics of repeat motifs frequency of classified repeat types.

Motif	Repeat motif								
Di	AG/TC (52.00%)	AC/TG (20.42%)	AT/TA (19.90%)	CG/GC (7.70%)	–	–	–	–	–	–
Tri	CCG/GGC (41.80%)	AGC/TCG (17.27%)	AGG/TCC (11.84%)	ACG/TGC (7.19%)	ACC/TGG (6.75%)	AAC/TTG (5.16%)	AAG/TTC (5.08%)	ATC/TAG (2.77%)	ACT/TGA (1.22%)	AAT/TTA (0.92%)

The results were very consistent with previous studies that the tri-nucleotide was the most abundance repeat among the types of SSRs (Kaur et al., 2012; Yates et al., 2014; Huang et al., 2016; Tian et al., 2017). In comparison to other nucleotide repeats, the tri-nucleotide expansions always lead to a homopolymeric amino acid run in an unaltered protein due to the retention of the original reading frame in translated regions (Kaur et al., 2012). However, the amplification of repeats other than tri-nucleotide is prevented in coding DNA, resulting in frame-shift mutations (Metzgar et al., 2000), which could be a possible explanation for the abundance of tri-nucleotide repeats. In the other hand, the AG/TC of di-nucleotide SSRs and CCG/GGC of tri-nucleotide SSRs were reported to be the largest number of motif in *Hemarthria* and grain crops (Temnykh et al., 2000; Kantety et al., 2002; Huang et al., 2016). The inverse GA/CT motif can describe multiple codons when transcribed into mRNA. Furthermore, a high proportion (8 and 10%) of amino acids Ala and Leu were translated from inverse GA/CT motif contributing it widely distribution across the genome (Kantety et al., 2002).

**FIGURE 1:**
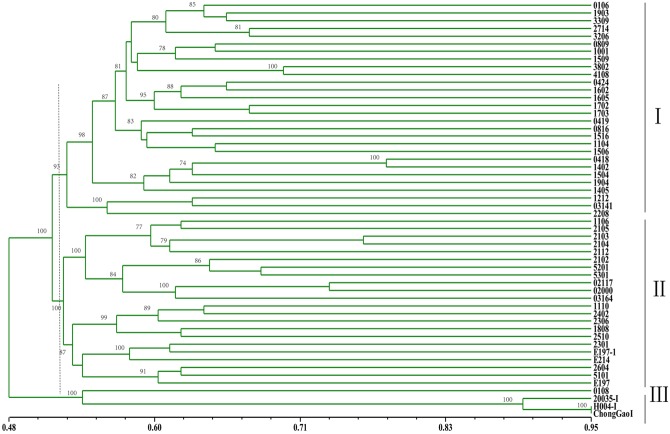
**Dendrogram of 51 *Miscanthus* and 3 *Hemarthria* genotypes based on genetic similarity (GS) coefficient**.

To validate the SSRs markers identified by RNA-seq, a total of 93 SSR primer pairs were randomly selected for validation including different motifs. Validation of these SSRs was based on the different size among 51 different *Miscanthus* genotypes and 3 *Hemarthria* species (Supplementary Table S1). In total, 70 (75.3%) SSRs were successfully amplified whereas 30 have polymorphic, including 9 for di-nucleotide, 16 for tri-nucleotide and 5 SSRs with motif lengths greater than 3 (**Table 7**). A total of 110 polymorphic loci generating from 30 SSRs were determined to be used for cluster analysis. The UPGMA dendrogram constructed by genetic similarity (GS) data obviously showed that three clusters were formed when the GS coefficient was 0.52. The group 1 accessions were mainly *Miscanthus sinensis* collected from the south of Sichuan and Chongqing which latitude was lower than N 31°. The *M. sinensis* genotypes of group 2 were primarily collected from the North of Sichuan and other genotypes of *M. floridulus*, *M. sacchariflorus*, *M. × giganteus* were also assigned into this groups. All three *Hemarthria* cultivars were classed to Group 3 as a foreign species (**Figure 1**). These results from the cluster analysis suggested that the *Miscanthus* SSR markers identified in this study have a high level of polymorphism clearly differentiating the *Miscanthus* species. More importantly, they had been proved having high transferability in the Andropogoneae tribe.

**Table 7:** Summary statistics of SSRs detection and validation in this study.

	Di	Tri	>3	Total
SSRs in the database	3,948	9,051	1,445	14,444
Ordered SSR primer pairs	19	60	14	93
Amplified SSRs	18	42	10	70
Polymorphic SSRs	9	16	5	30
Polymorphic rate	50.00%	38.10%	50.00%	42.86%

Previous studies on *Miscanthus* marker developing are limited to the number of available for use, and always markers from Tribe Andropogoneae (Poaceae) C4 crops such as sorghum (*Sorghum bicolor* L. Moench), maize (*Zea mays* L.), and sugarcane (*Saccharum officinarum* L.) were used for genetic studies (Ho et al., 2011; Kim et al., 2012; Chae et al., 2014; Yook et al., 2014; Nie et al., 2016). Although these large number of SSRs have been successfully applied to *M. sinensis* studies and proven with high transferability (Hernandez et al., 2001; Zhao et al., 2011; Xu et al., 2013; Chae et al., 2014; Yook et al., 2014; Nie et al., 2016), the specificity and function of markers seems less than satisfactory. The SSRs developed from *Miscanthus* transcriptome under drought conditions were aimed to the function regions among expressed unigenes, which could be facilitate better discovering of the marker-trait associations and higher efficiency.

### Population Structure and Association Analysis

**FIGURE 2:**
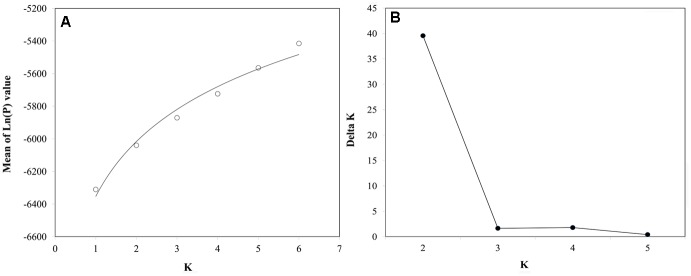
**Optimal value of *K* determined. (A)**, Mean log-likelihoods and their standard deviations from runs assuming different numbers of subpopulations (*K*). **(B)** Values of the delta *K* (Δ*K*), which tends to peak at the value of *K* that corresponds to the highest hierarchical level of substructure.

**FIGURE 3:**
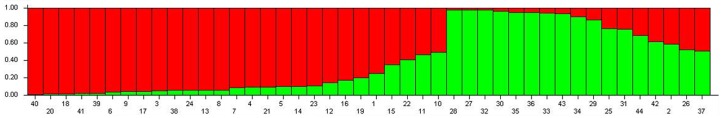
**Population structure analysis of 44 *Miscanthus* genotypes from southwest China.** Number 1-44 on the *x*-axis indicate the individual and number on the *y*-axis show the group membership.

Population structure of 44 genotypes was analyzed by using STRUCTURE V2.3.3 software. A total number of 110 filtered marker loci (by dropping the loci with minor allele frequency less than 5%) retained for structure and association analysis. Two optimum subgroups were determined by maximum likelihood and delta K (ΔK) values (**Figure 2**). Accordingly, 24 genotypes were assigned to group 1, 14 genotypes were to group 2, and 6 genotypes were referred not clearly assigned to the specific groups based on a significant membership threshold (*Q*-value) of 0.60 (**Figure 3**).

**FIGURE 4:**
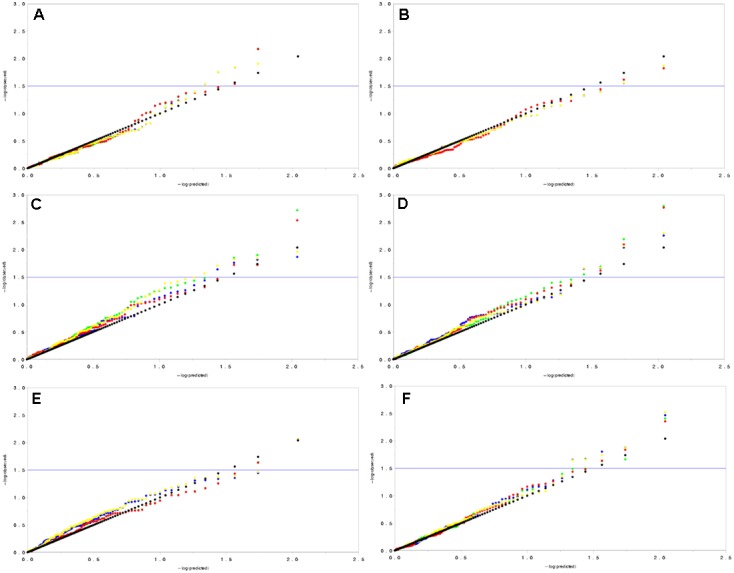
**Quantile–quantile plots for model comparison with physiological and photosynthetic traits under well water and drought treatment.** In this figure, black dots represent the standard line means that the predicted value equal to the observed value; yellow dots represent the simple linear model (without population structure and relative kinship); green dots represents the Q model; blue dots represents the K model; and red dots represent the Q+K model. **(A)** Evaluation of model types using markers for LWC. **(B)** Evaluation of model types using markers for MDA. **(C)** Evaluation of model types using markers for Pn. **(D)** Evaluation of model types using markers for Gs. **(E)** Evaluation of model types using markers for Ci. **(F)** Evaluation of model types using markers for Tn.

The association analysis was performed by taking Q (population structure) and K (relative kinship) into consideration using GLM and MLM model. Drought resistant related traits measured in this study under drought and control condition were used to test the model of Q, K, Q+K and simple linear model in QQ plots (**Figure 4**). Generally, in most conditions, the behavior of Q+K model was the best approximation to the excepted cumulative distribution of *P*-values. By implementing the Q+K models, a significant improvement was observed when compared with the other models. Based on Q+K model, a total of seven loci were detected significantly associate with RWC, Pn, Tn, Gs, and Ci (*P* < 0.01) under drought stress. Among them, 2 SSRs developing from CL12059.Contig2_All_5665_1 and CL10784.Contig3 _All_5453_1 were significantly associated with RWC, 1 SSRs from CL10592.Contig1_All_5404_1 associate with Pn and Gs simultaneously, 1 SSRs from CL11502.Contig2_All_5573_1 associate with Tn, 1 SSRs from CL10112.Contig3_All_5301_4 associate with Gs and 1 SSRs from CL10164.Contig1_All_5323_2 associate with Ci (**Table 8**). The contribution of single significant associated markers to the phenotypic variation was varied ranging from 11.89 to 25.30% (**Table 8**).

**Table 8:** Significant marker-trait association information of *Miscanthus* under drought stress.

Trait	Locus	Primer	Gene ID	*P*-value	*R*^2^ (%)
RWC-D	M92	SAUM-170	CL12059.Contig2_All_5665_1	6.15 E-04	25.30
RWC-D	M34	SAUM-73	CL10784.Contig3_All_5453_1	6.70 E-03	16.78
Pn-D	M25	SAUM-53	CL10592.Contig1_All_5404_1	2.90 E-03	14.79
Tn-D	M61	SAUM-121	CL11502.Contig2_All_5573_1	4.40 E-03	15.78
Gs-D	M25	SAUM-53	CL10592.Contig1_All_5404_1	1.70 E-03	21.32
Gs-D	M14	SAUM-10	CL10112.Contig3_All_5301_4	8.00 E-03	11.89
Ci-D	M2	SAUM-17	CL10164.Contig1_All_5323_2	8.70 E-03	14.67

Molecular markers have been widely used to analysis the genetic relationship of *Miscanthus* all around its distributed areas (Xu et al., 2013; Clark et al., 2014; Yook et al., 2014; Anzoua et al., 2015; Nie et al., 2016). Although some genetic maps (Kim et al., 2012; Ma et al., 2012; Swaminathan et al., 2012; Liu et al., 2015) and association mapping (Zhao et al., 2011; Slavov et al., 2014; Nie et al., 2016) with high density and resolution have been constructed, mapping of QTLs using the genetic map are limited owing to its absent of genomic information. Zhao et al. (2013) used 23 SSR markers transferred from *Brachypodium distachyon* to conduct marker-trait association analysis and 115 loci were generated. Among them, nine markers were detected to be significantly (*P* < 0.01) associated with heading date and biomass yield. Slavov et al. (2014) conducted genome-wide association studies in a 138 *M. sinensis* population by using single-nucleotide variants markers and a total of 17 significant associations were detected with phenology, morphology, and cell wall composition traits. Nie et al. (2016) used 104 pair of markers to conducted the marker-trait association analysis and among 1059 generated loci, 12 significant associations of biomass yield related traits were identified. In this study, among 110 loci generated from 30 randomly selected SSRs, 7 significant associations were identified with RWC and photosynthetic trait under drought stress indicating that the developed SSR markers have a relative stable linked efficiency to the drought tolerance related traits.

By searching for the function annotation of 7 unigenes accounting for the associated markers in the protein database (Supplementary Table S2), an definite answer was appeared. Especially, unigene CL10592.Contig1_All_5404_1 and CL10112.Contig3_All_5301_4 associated with Pn and Gs were annotated function related to vegetative cell wall protein, while unigene CL12059.Contig2_ All_5665_1 and CL10784.Contig3_All_5453_1 associated with RWC were annotated related to arginine/serine-rich protein and ubiquitin-like-specific protease. With the abundant genetic basis information, the developed markers could be potential candidates for improving *Miscanthus* selection for enhanced drought tolerance in a breeding program.

## Author Contributions

XiZ and LH conceived the project and designed the experiments; GN, LT, YZ, and XC performed the experiments; GN, XM, and XuZ analyzed the data; GN, LT, and LP finalized the manuscript.

## Conflict of Interest Statement

The authors declare that the research was conducted in the absence of any commercial or financial relationships that could be construed as a potential conflict of interest.

## Footnotes

1 http://trinityrnaseq.sourceforge.net/

2 http://pgrc.ipk-gatersleben.de/misa/

## Abbreviations

ABA: Abscisic acid
ANOVA: Analysis of variance
Ci: Intercellular CO_2_ concentration
DW: Dry weight
FW: Fresh weight
GLM: General linear model
GO: Gene ontology
Gs: Stomatal conductance
KEGG: Kyoto encyclopedia of genes and genomes
MAS: Marker-assisted selection
MCMC: Markov chain monte carlo
MDA: Malondialdehyde
MLM: Mixed linear model
NGS: Next-generation sequencing
NR: Non-redundant protein sequence
PAGE: Polyacrylamide gel electrophoresis
Pn: Net photosynthetic rate
RWC: Relative water content
SAHN: Sequential agglomerative hierarchical and nested clustering
SSRs: Simple sequence repeats
SW: Saturated weight
SWC: Soil water content
Tn: Transpiration rate
Swiss-Prot: Annotated protein sequence
UPGMA: Unweighted pair group method with arithmetic means
WUE: Water-use efficiency.
