# Supplementary material for: Development of SSR Markers Based on Transcriptome Sequencing and Association Analysis with Drought Tolerance in Perennial Grass Miscanthus from China
Source: Front Plant Sci. 2017 May 16;8:801. doi: 10.3389/fpls.2017.00801 (PMC5432562; doi:10.3389/fpls.2017.00801)
Supplement: Supplementary file 1 [file Table_1.DOCX]

Supplementary Material

**Transcriptome-Based Development of Molecular Markers and Association Analysis with Drought Tolerance in Perennial Grass *Miscanthus* from China**

**Gang Nie^1, †^, Lu Tang^1, †^, Yajie Zhang^1^, Linkai Huang^1^, Xiao Ma^1^, Xin Cao^1^, Ling Pan^1^, Xu Zhang^1^, Xinquan Zhang^1,^****^*^**

^1^ Department of Grassland Science, Animal Science and Technology College, Sichuan Agricultural University, Chengdu, Sichuan 611130, China

**^†^** These authors contributed equally. ^*^Corresponding author: Xinquan Zhang, Department of Grassland Science, Animal Science and Technology College, Sichuan Agricultural University, Chengdu, Sichuan 611130, China, *E-mail address:* zhangxq@sicau.edu.cn

# Supplementary Tables

**Supplementary Table 1** Geographic information for genotypes used in this study

| **NO.** | **Identity** | **Source*** | **Latitude (N)** | **NO.** | **Identity** | **Source** | **Latitude (N)** |
| --- | --- | --- | --- | --- | --- | --- | --- |
| 1 | 0106 | Laoban Mountain, Sichuan | N29°58′42.2″ | 28 | 2104 | JiangYou, Sichuan | N31°56′59.5″ |
| 2 | 0108 | Laoban Mountain, Sichuan | N29°58′43.1″ | 29 | 2105 | JiangYou, Sichuan | N31°59′45.6″ |
| 3 | 0419 | BiFengXia, Sichuan | N30°05′01.9″ | 30 | 2112 | JiangYou, Sichuan | N32°03′04.4″ |
| 4 | 0418 | BiFengXia, Sichuan | N30°05′01.9″ | 31 | 2208 | JiangYou, Sichuan | N32°04′26.4″ |
| 5 | 0424 | BiFengXia, Sichuan | N30°05′01.9″ | 32 | 2301 | Jian’Ge, Sichuan | N32°13′58.3″ |
| 6 | 0809 | ErlangMountain, Sichuan | N29°53′44.5″ | 33 | 2306 | Jian’Ge, Sichuan | N32°13′58.3″ |
| 7 | 0816 | ErlangMountain, Sichuan | N29°56′43.1″ | 34 | 2402 | GuangYuan, Sichuan | N32°38′16.9″ |
| 8 | 1001 | NibaMountain, Sichuan | N29°42′47.5″ | 35 | 2510 | GuangYuan, Sichuan | N32°38′59.5″ |
| 9 | 1104 | RenShou, Sichuan | N30°00′26.6″ | 36 | 2604 | GuangYuan, Sichuan | N32°39′16.5″ |
| 10 | 1106 | RenShou, Sichuan | N30°00′13.6″ | 37 | 2714 | DaYing, Sichuan | N30°36′36.7″ |
| 11 | 1110 | RenShou, Sichuan | N30°00′06.6″ | 38 | 3206 | BaNan, ChongQing | N29°31′10.7″ |
| 12 | 1212 | RenShou, Sichuan | N30°01′09.9″ | 39 | 3309 | NanChuan, ChongQing | N29°09′25.9″ |
| 13 | 1402 | HongYa, Sichuan | N29°50′19.1″ | 40 | 3802 | GuiYang, GuiZhou | N27°42′52.1″ |
| 14 | 1405 | HongYa, Sichuan | N29°50′26.8″ | 41 | 4108 | HuangGuoShu, GuiZhou | N25°58′22.5″ |
| 15 | 1504 | ZiZhong, Sichuan | N29°49′03.4″ | 42 | 5101 | YanTai, , Shandong | - |
| 16 | 1506 | ZiZhong, Sichuan | N29°49′03.4″ | 43 | 03164 | Hunan Agricultural University | - |
| 17 | 1516 | ZiZhong, Sichuan | N29°49′06.0″ | 44 | 03141 | Hunan Agricultural University | - |
| 18 | 1602 | LuZhou, Sichuan | N28°52′44.9″ | 45 | 02117 | Hunan Agricultural University | - |
| 19 | 1605 | LuZhou, Sichuan | N28°52′44.9″ | 46 | 02000 | Hunan Agricultural University | - |
| 20 | 1702 | LuZhou, Sichuan | N28°49′01.7″ | 47 | E197-1 | JiuJiang, JiangXi | - |
| 21 | 1703 | LuZhou, Sichuan | N28°49′01.7″ | 48 | E197 | JiuJiang, JiangXi | - |
| 22 | 1808 | YiBin, Sichuan | N28°45′08.8″ | 49 | E214 | JiuJiang, JiangXi | - |
| 23 | 1903 | YiBin, Sichuan | N28°45′31.0″ | 50 | 5201 | Shandong Agricultural University | - |
| 24 | 1509 | ZiZhong, Sichuan | N29°49′03.4″ | 51 | 5301 | Tai’An, Shandong | - |
| 25 | 2102 | JiangYou, Sichuan | N31°56′59.5″ | 52 | 20035-I | Ya’An, , Sichuan | - |
| 26 | 1904 | YiBin, Sichuan | N28°45′31.0″ | 53 | H004-I | GuangXi | - |
| 27 | 2103 | JiangYou, Sichuan | N31°56′59.5″ | 54 | ChongGao | Ya’An, , Sichuan | - |

^*^NO. 1 to 44 indicate *M. sinensis*; NO. 45 to 49 indicate *M. floridulus*; NO. 50 indicate *M. x giganteus*; NO. 51 indicate *M. sacchariflorus*; NO. 52 to 54 indicate *Hemarthria compressa.*
